# The use of 3D ceramic block graft compared with autogenous block graft for rehabilitation of the atrophic maxilla: a randomized controlled clinical trial

**DOI:** 10.1186/s13063-022-06843-3

**Published:** 2022-10-23

**Authors:** Carolina Mendonça de Almeida Malzoni, Victor Gonçalves, Juliana Possari, Elcio Marcantonio Junior

**Affiliations:** Department of Diagnosis and Surgery, School of Dentistry, São Paulo State University – UNESP, Araraquara, SP Brazil

**Keywords:** 3D-printed bone graft, β-tricalcium phosphate, Patient-specific bone graft, Atrophic maxilla, Synthetic bone substitute

## Abstract

**Background:**

Dental implant placement may require a bone graft for vertical and horizontal alveolar ridge augmentation. Due to its osteoconduction, osteoinductive, and osteogenesis, autogenous bone graft characteristics are considered the standard gold treatment. However, autografts can promote postoperative morbidity and implicate difficulties concerning the graft adaptation to the recipient's bone since it can eventually avoid gaps. To overcome these issues, this trial will compare the performance of Plenum^®^ Oss 3D_β_ fit, an alloplastic graft, and a 3D-printed patient-specific graft based on β-tricalcium phosphate to the autograft procedure.

**Methods:**

This is a split-mouth randomized clinical study designed to evaluate the performance of personalized (patient-specific) bioceramic bone grafts (Plenum^®^ Oss 3D_β_ fit) for bone augmentation of the atrophic anterior maxilla in comparison to the autogenous bone graft. We hypothesize that the gain and maintenance of the grafted area volume and the quality of the newly formed bone tissue after eight months postoperative with the synthetic patient-specific graft will be superior to the autogenous bone graft. To assess the quantity and the quality of bone neoformation, volumetric and histological analyses will be performed.

**Discussion:**

The fabrication of medical devices by additive manufacturing presents advantages over conventional manufacturing processes, mostly related to the precision of geometry and anatomy. Additionally, the osteoconductive proprieties of β-tricalcium phosphate enable this synthetic bone substitute as an alternative solution over autogenous graft for bone defect reconstruction. Thus, patient-specific bone grafts can potentially improve patient satisfaction, reducing the need for autogenous bone grafts, consequently avoiding implications related to this type of treatment, such as patient morbidity.

**Trial registration:**

This study is registered in REBEC (Registro Brasileiro de Ensaios Clínicos): RBR-76wmm3q; UTN: U1111-1272-7773. Registration date: 14 September 2021.

## Administrative information

Note: the numbers in curly brackets in this protocol refer to SPIRIT checklist item numbers. The order of the items has been modified to group similar items (see http://www.equator-network.org/reporting-guidelines/spirit-2013-statement-defining-standard-protocol-items-for-clinical-trials/)

**Table:** 

Title {1}	The use of 3D-ceramic block graft compared with intra-oral autogenous bone block graft for rehabilitation of the atrophic maxilla: a randomized controlled split-mouth clinical trial.
Trial registration {2a and 2b}.	REBEC (Registro Brasileiro de Ensaios Clínico): RBR-76wmm3q.
Protocol version {3}	08/January/2022, version 02
Funding {4}	Primary Sponsor: M3 Health Indústria e Comércio de Produtos Médicos, Odontológicos e Correlatos Sociedade Anônima, Jundiaí, Brazil. Secondary Sponsor: School of Dentistry, São Paulo State University (Unesp), Araraquara, Brazil.
Author details {5a}	Carolina Mendonça de Almeida Malzoni *Department of Diagnosis and Surgery, School of Dentistry, São Paulo State University – UNESP. Araraquara, SP, Brazil.* Victor Gonçalves *Department of Diagnosis and Surgery, School of Dentistry, São Paulo State University – UNESP. Araraquara, SP, Brazil.* Juliana Posssari *Department of Diagnosis and Surgery, School of Dentistry, São Paulo State University – UNESP. Araraquara, SP, Brazil.* Elcio Marcantonio Junior *Department of Diagnosis and Surgery, School of Dentistry, São Paulo State University – UNESP. Araraquara, SP, Brazil.*
Name and contact information for the trial sponsor {5b}	Sybele Saska Specian, sybele.saska@plenum.bio Thaís Cabrera Galvão Rojas, thais.rojas@plenum.bio
Role of sponsor {5c}	Primary sponsors (M3 Health) contributed to the study design and protocol elaboration. M3 Health and the Principal investigator team have decided to submit the written report for publication. The principal investigator (Elcio Marcantonio Junior) and the coordinating researcher (Carolina Mendonça de Almeida Malzoni) elaborated the study design and will be responsible for performing the trial. This research team will run the interventions, analyze the data, and publish the results.

## Introduction

### Background and rationale {6a}

The placement of dental implants in atrophic jaws is always a challenging condition. Before implant placement, bone grafts are frequently recommended for vertical and horizontal alveolar ridge augmentation. The autogenous bone graft stands out among other bone grafts due to its osteoconduction, osteoinductive, and osteogenesis properties. Although autogenous bone grafts are considered the gold standard for the reconstruction/replacement of bone grafts, the use of autografts is limited by several factors: mainly due to the significant postoperative morbidity of the patient and the insufficient amount of donor tissue [1, 2].

These disadvantages have led to new materials and promising methods for tissue repair, especially for bone tissue. In addition, homogenous and heterogenous bone grafts may promote immunogenicity, limiting these grafts’ use in specific surgical protocols [3]. Nevertheless, even with rigorous processes for deproteinization of these grafts, there is still a theoretical risk of disease transmission [1, 2, 4–6]. Alloplastic bone grafts, in turn, are synthetic biomaterials classified according to their origin. The alloplastic materials of ceramic sources mainly include calcium phosphate phases, which are essential mineral phases constituent of teeth and bones [4, 7]. Among ceramics, calcium phosphates are ceramics with Ca/P molar ratios ranging from 0.5 to 2.0 and can be found in various types, known as hydroxyapatite (HA) and β-tricalcium phosphate (β-TCP). HA and β-TCP are biocompatible materials used as excellent bone substitutes [5, 8] due to their osteoconductive properties and protein absorption capacity. However, bioceramics have different rates of in vitro solubility, which reflects in the in vivo degradation, i.e., the greater the Ca/P molar ratio lower the solubility of bioceramics; thereby HA phase has slower reabsorption, while the β-TCP has a faster reabsorption rate [4, 9].

Ceramic bone grafts can also be classified in their presentation, such as block or particulate grafts. When significant bone reconstruction in height and thickness are desired, and the bone defect does not have walls to favor bone regeneration, bone block grafts are indicated since they present satisfactory and predictable results for bone reconstruction/replacement [1, 2, 8, 10–12]. Indeed, the results obtained support the use of ceramic block grafts, proving to be an extremely beneficial technique, as it guarantees a safe, biocompatible bone substitute with unlimited availability. It also reduces the patient’s postoperative morbidity by not involving a second surgical site for bone graft removal. In addition, during the execution of the block graft technique, one of the most critical moments of the surgical procedure is the preparation of the graft for its adaptation to the recipient, which requires the experience from the surgeon to avoid gaps between the graft and host bone, reducing the chances of success [10–14]. This step can be facilitated by using ceramic bone grafts from 3D printing, a technology developed by tissue engineering that has contributed to the planning and execution of bone reconstructions [10, 13–15].

From initial cone-beam computed tomography (CBCT) scans and intraoral scanning, bone substitute scaffolds can be created and designed individually on the patient’s bone defect. In this way, the adaptation between them is guaranteed, and both patient and surgeon benefit by reducing the procedure's time and the patient’s morbidity [13–17]. Clinical studies using 3D ceramic scaffolds for bone regeneration have already been carried out and have shown satisfactory results [10, 13–15], which ensure their clinical applicability. However, the literature still lacks randomized clinical studies.

Plenum^®^ Oss 3D_β_ fit is a patient-specific medical device printed with custom dimensions. This medical device consists of a porous structure based on β-TCP (≥ 95% of β-TCP), planned by DICOM (Digital Imaging and Communications in Medicine) images from CBCT and manufactured by additive manufacturing technology (3D printing), producing personalized bone graft with complex geometrics. This bone substitute works like a bone graft, supporting the tridimensional bone reconstruction. Furthermore, the Plenum^®^ Oss 3D_β_ fit is slowly reabsorbed by the organism, favoring the substitution of the graft to the newly formed bone during the healing process or tissue regeneration.

Thus, this split-mouth randomized clinical study aims to evaluate the effect of bioceramic bone grafts in patient-specific blocks (Plenum^®^ Oss 3D_β_ fit) in bone augmentation of the atrophic anterior maxilla in comparison to the autogenous bone graft. The biocompatibility tests were performed following the normative instruction, ISO 10993-1 – *Biological Evaluation of medical devices*. The results showed that Plenum^®^ Oss 3D_β_ fit its safety with concerning its cytotoxicity and toxicity, endorsing the performance of this study in humans.

### Objectives {7}

The primary objective of this clinical investigation is to evaluate the increase/maintenance of bone graft volume after eight months postoperative when a synthetic patient-specific block graft is used compared to an intra-oral autogenous graft. The secondary objectives include the analysis and characterization of the new bone formation according to the graft used; Plenum^®^ Oss 3D_β_ fit, or autogenous; surgical particularities arising from the type of graft, such as the adaptation of the graft in the host bone; and operative time. In addition, implant stability and the adverse effects from both grafts and surgical procedures performed to install each kind of graft will also be analyzed.

### Trial design {8}

This protocol refers to a randomized superiority trial. This is a prospective, open, single-center, positive-controlled study, with two parallel groups, assigned as a split-mouth model, whereupon each side of the mouth will be allocated as an independent unit and each patient will receive both treatments, test and control.

## Methods: participants, interventions, and outcomes

### Study setting {9}

**Fig. 1:**
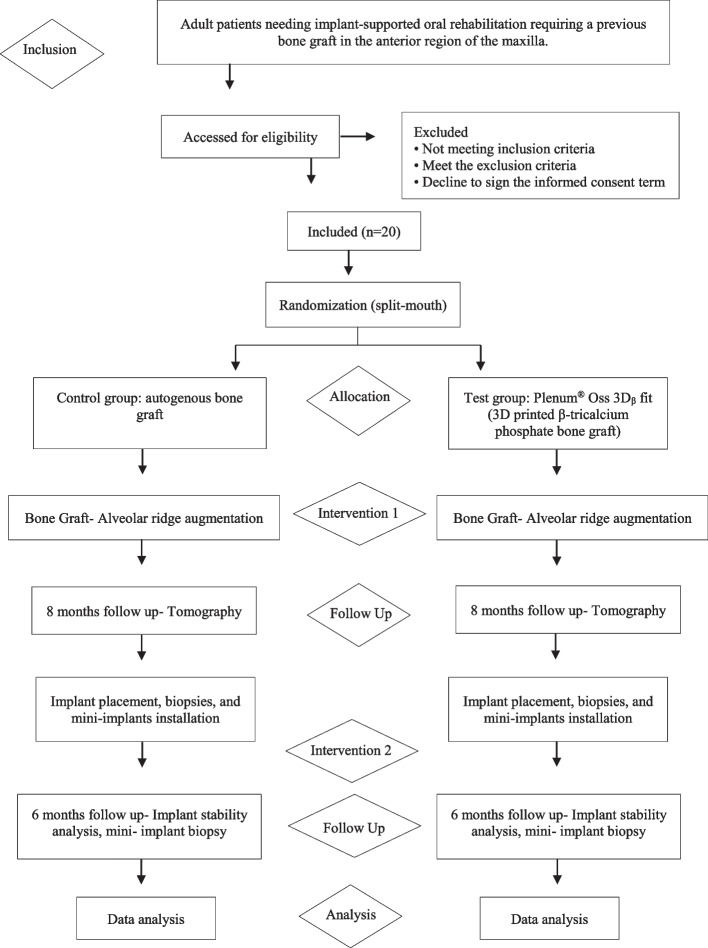
Flowchart of the study

The study will be performed with adult patients from the Implant Dentistry course at the School of Dentistry, São Paulo State University (Unesp), Araraquara, Brazil, looking for implant-supported oral rehabilitation and needing graft in the atrophic anterior maxilla (Fig. 1).

### Eligibility criteria {10}

To be enrolled in this study, patients must present bone insufficiency in the anterior region of the maxilla, with a minimum height of 7 mm and a width of less than or equal to 5 mm (assessed by CBCT), be over 18 (there are no age limit) years old, and have voluntarily signed the consent form. The exclusion criteria are patients who have general contraindications to the surgical procedure. These include patients who: are undergoing radiation in the head and neck region; have immunosuppression or immunodepression; are being treated or undergoing treatment with anti-resorptive drugs or drugs that alter bone metabolism (such as heparin, warfarin, cyclosporine, glucocorticoids, medroxyprogesterone acetate, cancer drugs, and thyroid hormone, prednisone, prednisolone, methylprednisolone, dexamethasone, cortisone, and triamcinolone); have an untreated periodontal disease; have poor oral hygiene; have uncontrolled diabetes; have psychiatric issues or unrealistic expectations; are pregnant (for women of childbearing potential is required contraception methods awareness); are lactating; and are smokers.

### Who will take informed consent? {26a}

The consent process will be done jointly by the principal investigator, or a member of his team already assigned to this task and the patient. The application of the term will be in the clinic, and in this consultation, participants will have the opportunity to ask questions, check visits, follow-up time, risks, and benefits of the study. In addition, the participants can take the term with them, to discuss with people they trust, if they wish. If the patient decides to participate in the study, the term must be rubricated by the responsible for the application and the patient, and be signed on the last page, always in two copies, one for the patient and another copy for being kept at the center until the end of the study. Upon completion of the study, the consent form will be stored by the sponsor.

### Additional consent provisions for collection and use of participant data and biological specimens {26b}

Not applicable; no ancillary studies are foreseen.

## Interventions

### Explanation for the choice of comparators {6b}

To allow the installation of dental implants in atrophic alveolar ridges, bone grafts must be previously performed for bone volume and height reconstruction. The autogenous graft stands out for its osteoconduction, osteoinductive, and osteogenesis properties, being considered the gold standard for this type of intervention [18].

### Intervention description {11a}

#### Tomography

All participants must undergo CBCT tests before the surgical procedure (T1), in post-surgical follow-up at 1 week (T2) and 8 months (T3).

#### Planning and production: patient-specific Plenum^®^ Oss 3D_β_ fit

A trained professional will perform the patient-specific block planning (Mimics and 3-Matic, Materialise, Belgium) from the DICOM files, manufactured by additive manufacturing in a 3D ceramic printer (CeraFab 7500 LITHOZ). The patient-specific (personalized) graft will be produced using Lithography-based Ceramic Manufacturing technology. The process involves printing the virtual model (previously designed and exported in an STL file) with β-TCP slurry. After finishing the printing process, the grafts will be cleaned with a solvent to remove excess resin inside the pores. Then, it will be sintered in a muffle furnace at 1000°C and sent for sterilization by gamma irradiation.

#### Preparation and installation of the autogenous bone graft and Plenum^®^ Oss 3D_β_ fit

The intra- and extra-oral asepsis will be performed with 0.12% and 2% chlorhexidine digluconate. Next, the local anesthesia will be applied with Articaine 4% 1:100.000 (Nova DFL, Brazil), followed by a linear incision over the alveolar crest and two vertical incisions. Next, the mucoperiosteal flap will be detached, and the autogenous bone graft collection will be started. A linear incision will also be made in the donor site, followed by a mucoperiosteal flap detachment and a mandible branch exposure. Trunk-conical drills and discs mounted in a straight handpiece will be used for osteotomy. After the graft removal, it will incubate in saline solution while the receptor site is prepared. Grooves will be performed in the receptor site to stimulate the vascularization, and the autogenous bone graft will be fixed in the anterior region of the maxilla by fixing screws. On the opposite side of the maxilla (test group), grooves will also be performed, and the 3D printed block will also be fixed by fixing screws. Then, a resorbable polydioxanone membrane, Plenum^®^ Guide (Plenum, Jundiaí, São Paulo, Brazil) will be used over 3 to 4 mm of the bone grafts, and the suture will be performed with Nylon 5.0 (Ethicon^®^, Jonhson & Jonhson, New Brunswick, Nova Jersey, EUA). All surgical procedures will be performed by the same professional.

#### Dental implants installation and biopsy

Eight months after the graft surgery, the patients will undergo implant placement surgery. The same aseptic care as described above will be performed. Following the local anesthesia, a linear incision will be made, a mucoperiosteal flap will be detached, and the fixing screws will be removed. Biopsies of the grafted areas will be obtained in the vestibular palatine sense direction [19] to involve the residual and new-formed bone without interfering with implant placement. The obtained biopsies will be immediately fixed in paraformaldehyde 4%. Then, the osteotomy sequence and implant placement will be performed, and the implants (Plenum, Jundiaí, São Paulo, Brazil) will be placed. The resonance frequency analysis (RFA) will measure the primary implant stability using Osstell^®^ (Integration Diagnostics AB, Goteborg, Switzerland). The measures will be performed on the mesial, distal, vestibular, and palatal regions. In the same surgical procedure, in the cases with high quantitative bone, mini-implants will also be used on the vestibule-palatine side for future histological analyses [19]. Finally, the suture will be performed using Nylon thread, Nylon Blue 5.0 (Techsuture^®^, Bauru, São Paulo, Brazil).

#### Healing caps placement

After 6 months of implant placement, healing caps will be placed to begin the prosthetic phase. The cover screw will be removed, RFA will check the secondary implant stability, and the healing cap installed. In the cases where mini-implants were used, they will be retrieved by trephine drills for posterior analysis [19].

### Criteria for discontinuing or modifying allocated interventions {11b}

Criteria were not applied because the study will evaluate a medical device in a split-mouth allocation; therefore, it is impossible to change the allocation once the device is installed. The patient will be discontinued from the study only if they experience a serious adverse event, which can be correlated with the device. So far, we have foreseen a serious allergic reaction to the device material and a serious infection.

### Strategies to improve adherence to interventions {11c}

Adherence will be maximized by educating patients about post-operative care and the importance of this process to the treatment successful.

### Relevant concomitant care permitted or prohibited during the trial {11d}

After the grafting procedure, the postoperative prescription will include Amoxicillin 875 mg + Potassium Clavulanate 125 mg every 12 h for 7 days, Nimesulide 100 mg every 12 h for 3 days, and Dipyrone 500 mg every 6 h for 3 days. Implant post-surgical medication will include Amoxicillin 500 mg every 8 h for 7 days, Nimesulide 100 mg every 12 h for 3 days, Dipyrone 500 mg every 6 h for 3 days, and chlorhexidine digluconate twice a day for 15 days.

Steroids and other drugs that interfere with calcium metabolism should not be administered during this clinical investigation, as described in the exclusion criteria.

### Provisions for post-trial care {30}

According to the Brazilian law (CNS Resolution No. 466 of 2012, items II.21 and IV.3.g), this trial will assure the participant and the companion of the reimbursement of expenses arising from the participation and the presence for consultations. Additionally, if any damage occurs to the participant due to the interventions from this research, the participant will be compensated and will receive full assistance for the necessary time (CNS Resolution No. 466 of 2012, items II.3.1 and II.3.2).

### Outcomes {12}

#### Primary endpoints

Evaluate the gain of increase/maintenance of the volume of the bone graft area and the quality of the newly formed bone tissue after eight months of the postoperative between the synthetic graft in a personalized block with the autogenous bone graft.

#### Secondary endpoints

The secondary endpoints are as follows:

- Assess bone neoformation in the anterior region of the maxilla when the personalized block graft is used compared to the use of autogenous bone block graft. This will be done by evaluating the percentage of new-formed bone tissue, soft tissue, and the rate of residual material from the bone graft
- Investigate whether the choice of bone graft interferes with the primary and secondary stability of implants installed in the region. Measures will be performed with RFA immediately after de implant placement and after 6 months
- Evaluate the influence of the graft on the maturation of newly formed bone tissue through the quantification of osteocalcin (OCN) from the biopsies retrieved just before the implant placement
- Assess the influence of the use of the graft type on the activity of osteoblastic cell differentiation through the quantification of morphogenetic protein-2 (BMP-2) from biopsies samples
- Evaluate the influence of autogenous and personalized graft on collagen matrix production through alkaline phosphatase quantification
- Investigate the influence of the graft in the differentiation of endothelial cells through the quantification of vascular endothelial growth factor (VEGF)
- Evaluate the volume of mineralized new-formed bone tissue through micro-tomographic examinations
- Verify the adaptation of the bone graft to the host bone
- Evaluate whether there is a difference in the operative time for performing the ceramic bone graft compared to the autogenous graft

### Study analysis

#### Volumetric analysis

Volume resorption will be evaluated by comparing the cone-beam computed tomography tests performed after (T2) the graft placement and eight months later (T3) with the software Horus Project®. In addition, from the CBCT T2 test, an analysis regarding the adaptation of the bone graft to the residual bone will be performed in both groups.

#### Biopsies analyses

##### Histological analysis

The obtained biopsies will be immediately stored in paraformaldehyde 4% for 48h and after immersed in 70° alcohol solution for the micro-tomographic test. Then, the biopsies will be washed with running water for subsequent immersion in EDTA solution, which will be changed three times a week for 50 days. After evaluating the correct decalcification of the biopsies, the dehydration process will be carried out. Dehydration will be done in increasing alcohol baths (70°, 90°, absolute alcohol), allowing the piece to be finally cleared in xylene for 3 h and embedded in paraffin.

The mini-implant biopsies will be washed with running water for 6 hours and then dehydrated in a growing series of ethanol (60–100%) and infiltrated and polymerized in light-curing resin (Technovit 7200 VLC, Kulzer Heraeus GmbH & CO, Wehrheim, Germany).

##### Micro-tomographic analysis

Before starting the process for inclusion in paraffin, the biopsies will be scanned by a microtomography (Skyscan®, Aatselaar, Belgium. Camera Pixel: 12.45; X-ray tube power: 65 kVP, X-ray intensity: 385 μA, integration time: 300 ms, filter: Al-1 mm and voxel size: 18 μm3c). Then, the images will be reconstructed, spatially repositioned, and analyzed by specific software (NRecon, Data Viewer, CTAnalyser, Aatselaar, Belgium). The threshold used in the analysis will be 25–90 shades of gray, and the values of mineralized tissue volumes will be obtained as a percentage. A trained examiner blinded to the experimental groups will perform this analysis.

##### Histomorphometric analysis

The paraffin blocks will be cut in serial cuts with 4μm thick along their entire length. To obtain the histological blades, the pattern of selecting the center cut of the block will be followed. Blades will be stained with hematoxylin and eosin (HE, Merck & Co. Inc., New Jersey, USA). Blades images will be captured and scanned using an optical microscope with fourfold magnification objectives and 5-fold magnification eyepieces (Diastar - Leica Reichert & Jung products, Germany), with a digital camera (DFC-300-FX, Leica Microsystems, Germany) with 1.3-megapixel resolution coupled to this microscope and connected to a microcomputer with a digitalized image analyzer software Image J 1,45 (Wayne Rasband National Institutes of Health, USA). The following parameters will be evaluated: newly formed bone percentage, soft tissue percentage, and residual bone graft percentage. Measurements will be performed by a single calibrated examiner blinded to experimental groups.

The mini-implant blocks with bone tissue will be cut at a central point using a cut and wear system (Exakt Apparatebeau, Hamburgo, Germany). The sections will be approximately 45 μm thick, stained with Stevenel’s blue associated with acid fuchsin, and analyzed under an optical microscope (DIASTAR – Leica Reichert & Jung products, Wetzlar, Germany) at × 100 magnification. Histomorphometric evaluations will be performed with the image analysis software (Image J, San Rafael, CA, USA). In addition, the percentages of bone-implant contact (%BIC) and the bone area between turns (%BBT) will be evaluated separately in the region between bone tissue and the center of the mini-implant. A blind and trained examiner will perform these analyses.

##### Immunohistochemical analysis

Histological sections from the same paraffin blocks used for the histomorphometric analysis will be mounted on blades with an appropriate glass surface (Fisher Superfrost Plus; Thermo Fisher Scientific - Waltham MA, USA). The immunohistochemical reaction will be performed using the indirect immunoperoxidase technique with an amplifier. Therefore, 3% hydrogen peroxide will be used (Merck Laboratories, Kenilworth, Nova Jersey, USA) to inhibit endogenous peroxidase. Antigen recovery will be performed by immersing the slides in citrate phosphate buffer, pH = 6, kept in a steamer (moist heat) for 20 min. Blocks of nonspecific reactions will be performed with bovine albumin (Sigma, San Luis, Missouri, USA) and skim milk. The primary antibodies against osteocalcin, alkaline phosphatase, VEGF, and BMP2 (Santa Cruz Biotechnology, Dallas, TX, EUA) are bone metabolism markers. BMP2 is one of the main proteins related to bone neoformation and development, while alkaline phosphatase and osteocalcin allow the evaluation of collagen matrix production and bone mineralization. Vascular endothelial growth factor (VEGF) enables the assessment of local angiogenesis. As a secondary antibody, the biotinylated anti-goat antibody produced in rabbits will be used (Pierce Biotechnology, Waltham, Massachusetts, USA); the amplifier will be Avidin and Biotin (Vector Laboratories, Burlingame, California, USA), and diaminobenzidine (DakoCytomation, Carpinteria, California, USA) will be used as the chromogen. At the end of the revelation by diaminobenzidine, the counterstaining of the histological sections will be performed with Harris hematoxylin. The sections will be analyzed under an optical microscope (LeicaR® DMLB, Heerbrugg, Switzerland) as well as the expression of proteins, coupled to an image capture camera (Leica^®^ DFC 300FX, Leica microsystems, Heerbrugg, Switzerland) and connected to a microcomputer with scanned image analyzer software (Leica Camera Software Box^®^, Leica Imaging Manager -IM50 Demo Software).

#### Implant stability analysis

The primary and secondary implant stability will be analyzed using the Osstell^®^ device (Osstell AB, Gothenburg, Sweden). This device determines the implant stability by the resonance frequency analysis. The system includes using a SmartPeg fixed to the implant through an integrated screw. The SmartPeg is excited by a magnetic impulse from the measuring probe of the portable instrument, and the implant stability coefficient (ISQ) is calculated from the response signal. Results are displayed on the instrument ranging from 1 to 100. The higher the ISQ number, the greater the stability of the implant.

Stability measurements will be obtained in two directions: buccal-palatal and mesiodistal. Values will be compared within groups about primary and secondary stability. They will also be compared between groups to assess whether there is a difference between implant stability in the test and control groups.

### Participant timeline {13}

**Table:** 

	Study period				
	Enrollment	Allocation	Post allocation		
Timepoint	− ***T***_**1**_	T0	T1 (intervention)	T2 (8-month follow up)	T3 (14-month follow up)
Patients visits	x				
Eligibility screen	x				
Informed consent	x				
Randomization: split-mouth		x			
**Intervention**					
Tomography (T1, T2, and T3)	x		x	x	
Graft installation			x		
Implant placement and biopsies				x	
Healing caps placement					x
**Assessments**					
Surgery analysis			x		
Implant stability				x	x
Biopsies				x	
Biopsies analyses				x	x
Adverse events			x	x	x

### Sample size {14}

Sample calculations were performed based on tomographic bone formation data from the study, which evaluated the volumetric stability of autogenous and allogeneic corticomedullary block grafts used for volume increase associated with edentulous alveolar ridges [19]. This choice was due to the lack of studies comparing β-tricalcium phosphate blocks and autogenous bone and the similarity of the methodology for evaluating the stability of block grafts. As a result, it was verified that the minimum difference between the means of treatments concerning the percentage of volumetric reduction of the graft was 11.90, with a standard deviation of 12.09. Therefore, considering a study power (1-β) of 0.8 and an *α* power of 0.05, a minimum sample of 20 participants was determined, each of whom will receive two grafts, one synthetic in a personalized block and another in an autogenous block. To maintain the power of the study, the sample size should be maintained, thus if any participant dropout or is discontinued before the analysis of the primary outcome, a new participant will be recruited instead, until complete 20 participants.

### Recruitment {15}

Recruitment will be performed by local radio and social media (Facebook). The advertisement will invite patients who need implant rehabilitation on both sides of the maxilla, and after the patients attend a screening visit, the inclusion criteria will be assessed. Additionally, as the study will be carried out at a public university that serves patients free of charge in various undergraduate and graduate courses, the study will be disseminated to students and professors, allowing them to refer patients with the necessary inclusion criteria to be enrolled in this study.

## Assignment of interventions: allocation

### Sequence generation {16a}

The treatments will be randomized using 20 binary sets (0- control treatment: autogenous graft and 1- test treatment: Plenum^®^ Oss 3D_β_ fit). The sets, 1 to 20, will correspond to each patient according to the enrolment order. The binaries numbers 0 and 1 will be randomized, corresponding to the treatment that will be applied. From left to right, the first number will define the treatment of the right side of the maxilla, and the second number will determine the treatment which will be applied on the left side.

### Concealment mechanism {16b}

Randomization of treatments will be performed using the ResearchRandomizer software (https://www.randomizer.org/).

### Implementation {16c}

The trial sponsor and the Principal investigator team will generate randomization with the allocation sequence.

## Assignment of interventions: blinding

### Who will be blinded {17a}

Not applicable; this is an open trial.

### Procedure for unblinding if needed {17b***}***

Not applicable; this is an open trial.

## Data collection and management

### Plans for assessment and collection of outcomes {18a}

For security, CT scans will be saved on the M3 Health’s server, which already has a security protocol. The biopsies analysis will be performed with positive controls for the antibody reactions.

Data management will follow the same procedures for verification, validation, and security of electronic systems used by the sponsoring company, M3 Health.

### Plans to promote participant retention and complete follow-up {18b}

Data from participants which have already been collected will be used in the analysis referring to the completed steps, even if they do not conduct the clinical investigation.

### Data management {19}

Data will be maintained at Electronic File Management System (SharePoint platform, Microsoft 365) and organized by the study sponsor. The hardcopy source documents will be stored at M3 Health in an appropriate room with controlled access. Patients’ identity will be determined by a letter/number code composed of the first and last letter of the name, the first and last letter of the last name, and the birth year. The CRFs will be transcribed to electronic forms, after the patient’s visit by the Ph.D. students, as a backup measure and also to report adverse events to the sponsor. These forms will be prepared by the sponsor, and after they are filled in, they will be saved, in a pdf file, on the company's server.

### Confidentiality {27}

According to the current legislation regarding General Law of Data Protection (LGPD), number: 13.709/2018.

### Plans for collection, laboratory evaluation, and storage of biological specimens for genetic or molecular analysis in this trial/future use {33}

Not applicable; the study will not collect biological specimens for genetics or molecular analyses.

## Statistical methods

### Statistical methods for primary and secondary outcomes {20a}

Data from this study will be analyzed for their distribution by the Shapiro-Wilk normality test. If the data are distributed according to normality, parametric tests will be used for the inferential analysis of the data. The comparison between groups in each experimental period will be performed using the unpaired *t*-test. The longitudinal evaluation of these data within each group will be evaluated using the ANOVA test for repeated samples complemented by the Tukey test. If the data are not distributed according to normality and the qualitative parameters, they will be analyzed inferentially using non-parametric statistical tests. The comparison between groups in each experimental period will be performed using the Mann-Whitney test. The longitudinal evaluation of these data within each group will be evaluated using the Friedman test complemented by the Dunn test. The BioStat software version 5.3 will be used to perform the statistical analysis of this project.

### Interim analyses {21b}

The principal investigator will decide to suspend the study if any serious adverse event is reported until the cause is identified. If it is verified that the event was not related to the medical device, the study will restart under sponsor permission. However, if the cause of the serious adverse event is related to the investigational device and the investigation is terminated, the interim results will be accessed by the principal investigator, that will perform the correspondent analysis.

### Methods for additional analyses (e.g., subgroup analyses) {20b}

Not applicable; all the analyses are already described in the study protocol.

### Methods in analysis to handle protocol non-adherence and any statistical methods to handle missing data {20c}

This study needs 20 participants to conclude the statistical analysis of the primary endpoint. Then, a new participant will be recruited whenever there is any dropout or loss of follow-up until the collection of the biopsy of 20 participants.

### Plans to give access to the full protocol, participant level-data, and statistical code {31c}

Not applicable; only the principal investigator, the research team, the sponsor, and the Brazilian National Health Surveillance Agency (ANVISA) will access participant-level datasets and statistical code.

## Oversight and monitoring

### Composition of the coordinating center and trial steering committee {5d}

The communication will be directly between the sponsor team, the principal investigator, and the coordinating investigator via telephone, e-mails, and internet meetings, following sponsor security protocol. A trial steering committee composed of the sponsor scientific board will constantly follow the trial progress by the electronic CRFs answers.

### Composition of the data monitoring committee, its role and reporting structure {21a}

The study monitoring will be performed by the sponsor, during 3 moments of the study: in the initiation, during the test device installation stage, and during the dental implant installation phase. In addition, an audit visit will be carried out shortly after the installation of the devices to verify data accuracy, completeness, traceability, and delays.

### Adverse event reporting and harms {22}

Adverse effects will be registered in the case report form and by phone call if the event were classified as serious by the principal investigator. According to the severity level of the event, the necessary actions will follow RDC 548/2021 of Brazilian Health Regulatory Agency (ANVISA) [20] obligations and recommendations.

### Frequency and plans for auditing trial conduct {23}

The audit monitoring visit will be conducted by the sponsor at least once during the clinical investigation, in the stitch removal phase after graft installation (3rd visit of the participants) or whenever the sponsor understands that there are severe or repeated Protocol deviations, or when there is suspicion of fraud when requested or suggested by a regulatory authority.

### Plans for communicating important protocol amendments to relevant parties (e.g., trial participants, ethical committees) {25}

Protocol amendments will be communicated to ANVISA. If they are substantial and directly reflect on subjects’ participation, they will be resubmitted to the same ethics committee that approved the study and then submitted to ANVISA for protocol amendment. Non-substantial amendments will be reported in the annual report which must be submitted to ANVISA.

### Dissemination plans {31a**}**

An annual report will be elaborated and submitted to ANVISA. After data analysis, the research will publish the results in an appropriate indexed life science journal. Participants will be communicated the results via e-mail or WhatsApp.

## Discussion

A range of particulate bone grafts is currently available on the market, which is clinically effective and predictable. However, the granules have limitations for adequate rehabilitation/reconstruction of the anatomical structure for complex geometry reconstructions. These bone reconstructions related to complex geometries sometimes require more than one surgical procedure to achieve the desired bone reconstruction: consequently increasing the cost of patient rehabilitation and often delivering a limited result. In the case of extensive reconstructions of bone volume, these grafts need to be associated with screws, meshes, and membranes.

Synthetic grafts are usually ceramic based on calcium phosphates, such as HA, β-TCP, or an association of both [21]. The β-TCP has been highlighted as a material of bone graft substitutes due to its resorption by cells, usually osteoclasts, which end up causing slight local acidification, which leads to the dissolution of β-TCP [22–24]. This process makes the β-TCP a resorbable compound, allowing a rapid replacement by new bone [23, 25, 26]. In addition, bioceramic β-TCP can be obtained through various production methods. One of these processes, additive manufacturing or 3D printing, has been drawing attention thanks to numerous advantages arising from the production method.

Applications of the additive manufacturing process in medicine have been reported since 1994, which have led to the development of personalized implants for the precise reconstruction of bone structure [27, 28]. There are several advantages of additive manufacturing over conventional manufacturing processes, such as control of the porosity and roughness of the blocks (printed parts), complete control over geometry, the possibility of creating structures with interconnected pore systems, precise details of anatomy, low material waste, production of several pieces at once, ability to create sophisticated details and complex internal geometries, high design fidelity, process reliability, and high reproducibility [27–29]. Thus, osteoconductive grafts manufactured by additive manufacturing may provide an alternative solution for the reconstruction of significant bone defects and rehabilitation of patients with severe craniofacial bone atrophy, such as early dental loss, congenital malformation, or after resection of cysts and tumors [30]. Clinical studies using 3D ceramic frameworks for bone regeneration have already been carried out and have shown satisfactory results (9,10,12,14), which ensure their clinical applicability. Furthermore, a recent systematic review [31] evaluated the effectiveness of synthetic blocks for bone augmentation in preclinical studies. As a result, the authors concluded that synthetic blocks are a viable alternative for bone regeneration. However, more clinical studies need to be conducted to confirm the effect of these synthetic blocks on bone neoformation. Thus, the protocol presents a randomized split-mouth clinical study that aims to evaluate the performance and safety of block ceramic bone grafts composed of patient-specific β-TCP for bone augmentation of anterior maxillae.

### Trial status

This is the second version of the protocol. This protocol is approved by ANVISA (for regulatory compliance), and the study will start to recruit the participants in February.

## Data Availability

The sponsor, principal investigator, the research team, and ANVISA (Brazilian Health Regulatory Agency) will access the final data set.

## Abbreviations

β-TCP: β-tricalcium phosphate
ANVISA: National Health Surveillance Agency (Brazilian Health Regulatory Agency)
BBT: Bone area between turns
BIC: Bone-implant contact
BMP-2: Morphogenetic protein 2
CBCT: Cone beam computed tomography
CONEP: National Research Ethics Commission
DICOM: Digital Imaging and Communications in Medicine
HA: Hydroxyapatite
ISQ: Implant stability coefficient
LGPD: General Law of Data Protection
OCN: Osteocalcin
REBEC: Brazilian Clinical Trials Registry
RFA: Resonance frequency analyses
VEGF: Vascular endothelial growth factor
UNESP: São Paulo State University
